# Molecular and temporal control of restimulation-induced cell death (RICD) in T lymphocytes

**DOI:** 10.3389/fceld.2023.1281137

**Published:** 2023-10-30

**Authors:** Katherine P. Lee, Benjamin Epstein, Camille M. Lake, Andrew L. Snow

**Affiliations:** Department of Pharmacology and Molecular Therapeutics, Uniformed Services University of the Health Sciences, Bethesda, MD, United States

**Keywords:** apoptosis, T cell receptor, RICD, ALPS, XLP-1, metabolism, CWID

## Abstract

For effective adaptive immunity, T lymphocytes must rapidly expand and contract in an antigen-specific manner to effectively control invading pathogens and preserve immunological memory, without sustaining excessive collateral damage to host tissues. Starting from initial antigen encounter, carefully calibrated programmed cell death pathways are critical for maintaining homeostasis over distinct phases of the T cell response. Restimulation-induced cell death (RICD), a self-regulatory apoptosis pathway triggered by re-engagement of the T cell receptor (TCR), is particularly important for constraining effector T cell expansion to preclude overt immunopathology; indeed, genetic disorders affecting key molecules involved in RICD execution can manifest in excessive lymphoproliferation, malignancy, and autoimmunity. Herein we review our current knowledge of how RICD sensitivity is ultimately regulated over the course of an immune response, including recent revelations on molecules that tune RICD by enforcing resistance or promoting susceptibility in expanding versus mature effector T cells, respectively. Detailed dissection of the molecular and temporal control of RICD also illuminates novel therapeutic strategies for correcting abnormal T cell responses noted in various immune disorders by ultimately tuning RICD sensitivity.

## Introduction

The fundamental charge of the immune system is to properly discriminate “self” from “foreign,” tolerating the former and reacting only to the latter in order to neutralize, isolate and/or eliminate countless pathogens without inadvertently causing damage to the body itself. Acquired or adaptive immunity relies upon the rapid expansion of antigen-specific B and T lymphocytes to secrete cytokines, generate high-affinity antibodies, and mount cytotoxic responses against infected target cells. Effective recruitment and expansion of antigen-specific cells is achieved via “clonal selection,” a theory first postulated by Nobel laureate Dr. Frank Burnett well before the discovery of B and T cell receptors (BCR/TCRs) constructed via somatic gene recombination (Burnet, 1976). By generating and maintaining a vast population of lymphocytes comprising a repertoire of antigen receptors unique to each individual cell, highly specific adaptive immunity to myriad microbial challenges is ensured upon *antigen-directed* selection of specific clones. Developing T cells must be “educated” to properly recognize antigens in the form of peptides presented by a set of germline-encoded, polymorphic major histocompatibility (MHC) molecules. MHC restriction means that T cell receptors (TCRs) are inherently self-reactive to some degree: indeed, thymocytes harboring a TCR incapable of binding to self-MHC will fail positive selection and undergo death by neglect. By contrast, overtly self-reactive T cells expressing a TCR with high affinity for self-peptide/MHC complexes are destined for the regulatory T cell (Treg) compartment, or more often deleted by negative selection, a TCR-induced apoptosis program at the heart of “central tolerance” (Hogquist et al., 2005). However, this process has evolved to be imperfect, accommodating some degree of self- and cross-reactivity that ostensibly affords flexibility in building a diverse yet finite repertoire of TCRs capable of mounting an effective response to almost any foreign protein antigen. Several additional mechanisms of “peripheral tolerance” have evolved to prevent mature, self-reactive peripheral T cells from triggering autoimmunity, including anergy, FOXP3^+^CD4^+^ regulatory T cell (Tregs), exhaustion/senescence, and clonal deletion via apoptosis (ElTanbouly and Noelle, 2021). Only when combined with appropriate costimulation dictated by innate immune-dependent sensing of “danger” signals, lymphocyte clones can rapidly proliferate and acquire effector functions to effectively combat an infectious challenge (Matzinger, 2001). The potential for tissue damage and immunopathology is never eliminated, however, especially in light of some inherent self- and cross-reactivity built into the TCR repertoire.

Although several flavors of programmed cell death have been illuminated in recent years, apoptosis remains the most important, non-inflammatory form of programmed cell death responsible for maintaining tissue homeostasis throughout the body. Indeed, lymphocytes circulating in blood, lymphoid tissues and everywhere in between are no exception. As our genetic and molecular understanding of apoptosis has progressed from original discoveries in *C. elegans* to detailed studies of mammalian cells, we now recognize two distinct forms of apoptosis: extrinsic and intrinsic (Lossi, 2022). Extrinsic apoptosis is prompted by engagement of dedicated death receptors (DRs) of the tumor necrosis factor receptor superfamily (e.g., FAS/CD95) expressed on the plasma membrane. Ligation of pre-assembled DRs initiates the recruitment of adaptor proteins (e.g., FADD) and “initiator” caspase zymogens (pro-caspase 8/10) that assemble into a death-inducing signaling complex (DISC), a platform for caspase 8/10 dimerization and cleavage to generate proteolytically active caspase 8/10. By contrast, genotoxic insults or withdrawal of growth factors can trigger the intrinsic apoptosis pathway, for which the relative balance of pro- and anti-apoptotic BCL-2 family proteins ultimately controls mitochondrial depolarization (via pores formed by pro-apoptotic BAX and BAK) and release of messengers like cytochrome C. The “apoptosome,” comprised of cytochrome c, APAF-1 and pro-caspase 9, serves a parallel platform function as the DISC to produce active caspase 9. Active initiator caspases (8/9/10) converge on the activation of “executioner” caspases (3/6/7) that subsequently cleave protein targets associated with hallmarks of apoptotic death, including DNA fragmentation and chromatin condensation, membrane blebbing, and exposure of key “eat me” signals that ensure prompt removal of dead cells/debris by phagocytes. Depending on cellular context and relevant stimuli, crosstalk and cooperation exists between extrinsic and intrinsic pathways to properly regulate cellular turnover and safeguard tissue homeostasis. As we focus on T cell homeostasis herein, myriad prior reviews discuss the molecular mechanisms of both pathways in greater detail (Lenardo et al., 1999; Green et al., 2003; Marrack and Kappler, 2004; Strasser and Pellegrini, 2004; Arnold et al., 2006; Brenner et al., 2008; Snow et al., 2010; Zheng et al., 2017).

**FIGURE 1:**
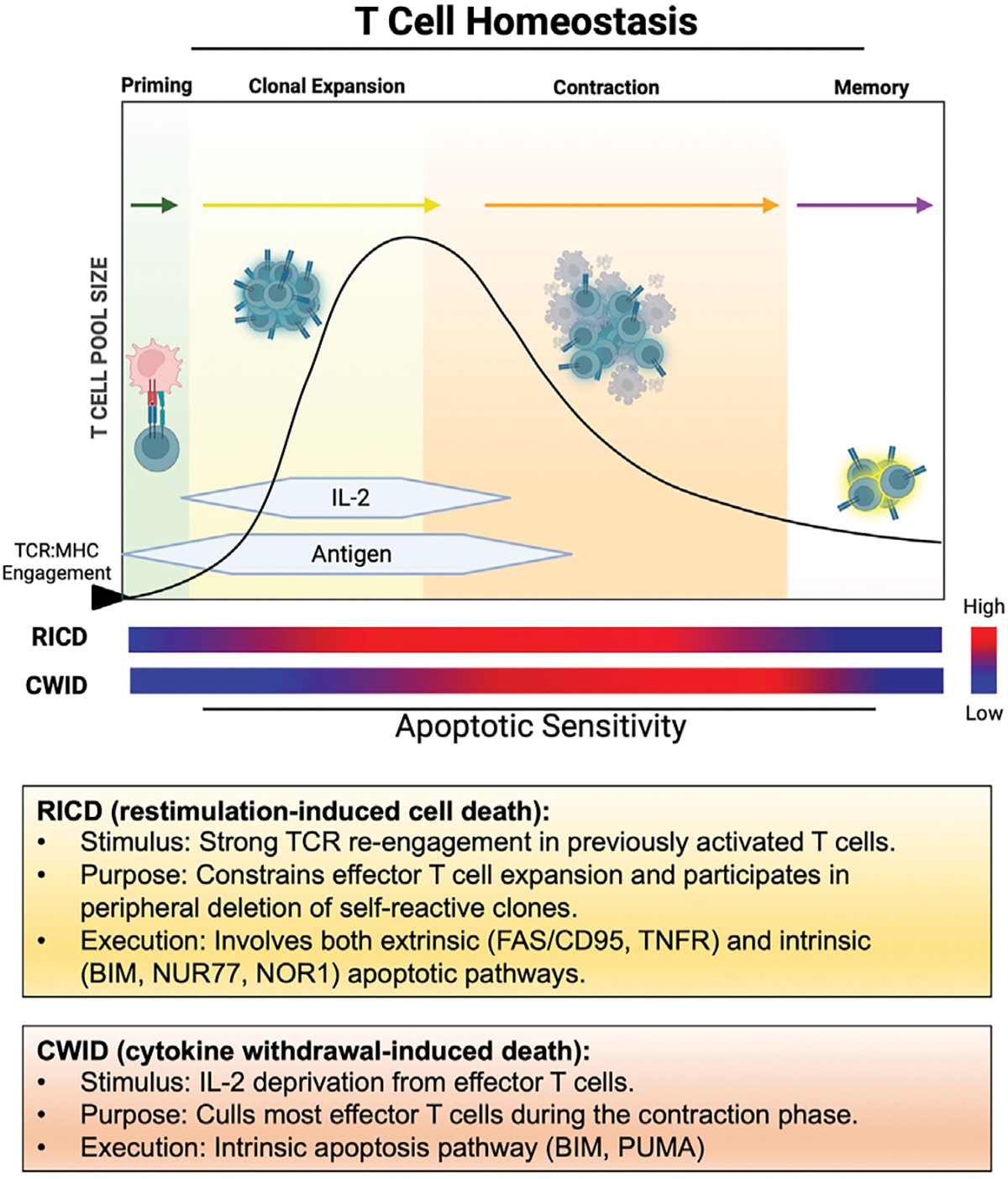
Temporal windows of apoptosis sensitivity during the T cell response. After initial engagement with pMHC, naïve/resting T cells undergo clonal expansion in the presence of abundant antigen and IL-2, remaining relatively resistant to RICD over several rounds of division. RICD sensitivity increases over time to help restrain excessive effector T cell accumulation. As antigen is cleared and IL-2 production wanes, most effectors undergo contraction via CWID, leaving a small pool of memory T cells to survive for future antigen encounters.

In contemplating the tenets of clonal selection theory and how the T cell repertoire is ultimately constructed, it makes sense that the fate of any T cell clone is inextricably tied to antigen recognition through the TCR, linked from early development in the thymus to the terminal stages of an effector T cell response in the periphery. Initial T cell activation requires two signals: a robust TCR signal (“signal 1”) bolstered quantitatively and qualitatively by costimulation (e.g., via CD28, “signal 2”), enabling transcription of IL-2 and its high affinity receptor (IL-2Rα/CD25) to fuel rapid clonal expansion via autocrine/paracrine IL-2R signaling. As effector T cell proliferation and differentiation proceeds, two major apoptotic programs shape the magnitude of the responding effector T cell pool for the duration of the response: cytokine withdrawal-induced death (CWID) and restimulation-induced cell death (RICD) (Brenner et al., 2008; Snow et al., 2010; Zheng et al., 2017). Indeed, the relative abundance of IL-2 and antigen in the local environment (i.e., TCR engagement over time) dictates when and how these two mutually exclusive apoptosis pathways come into play (Figure 1).

As foreign antigen is eliminated and cytokine production declines, the vast majority of terminally differentiated effector T cells are culled by CWID, an exclusively intrinsic apoptotic program. CWID governs most of the effector T cell contraction phase at the conclusion of an adaptive immune response, with only a small population of memory T cells preserved to enable a rapid recall response upon a second antigen encounter. In particular, loss of IL-2 signaling induces expression of the pro-apoptotic “BH3-only” protein BIM (with a minor role for PUMA as well) (Erlacher et al., 2006). As BIM accumulates and overwhelms the pool of anti-apoptotic proteins protecting the mitochondria from BAX/BAK-mediated pore formation (e.g., BCL-2, BCL-xL, MCL-1), CWID commences with loss of mitochondrial membrane potential, apoptosome activation, and apoptosis. Although the vast majority of CWID occurs during effector T cell contraction, this pathway is also at play in FOXP3^+^ Treg-dependent suppression of self-reactive T cells. Tregs are completely reliant on “stealing” IL-2 from conventional T cells via high constitutive expression of CD25. While Tregs are equipped with several molecular tools for modulating conventional T cell activation (Pandiyan and Lenardo, 2008), competitive consumption of IL-2 may be the most important mechanism by which Tregs prevent the expansion of self-reactive T cells by inducing CWID, confirmed both *in vitro* and *in vivo* (Pandiyan et al., 2007; Liu et al., 2015; Wong et al., 2021).

**FIGURE 2:**
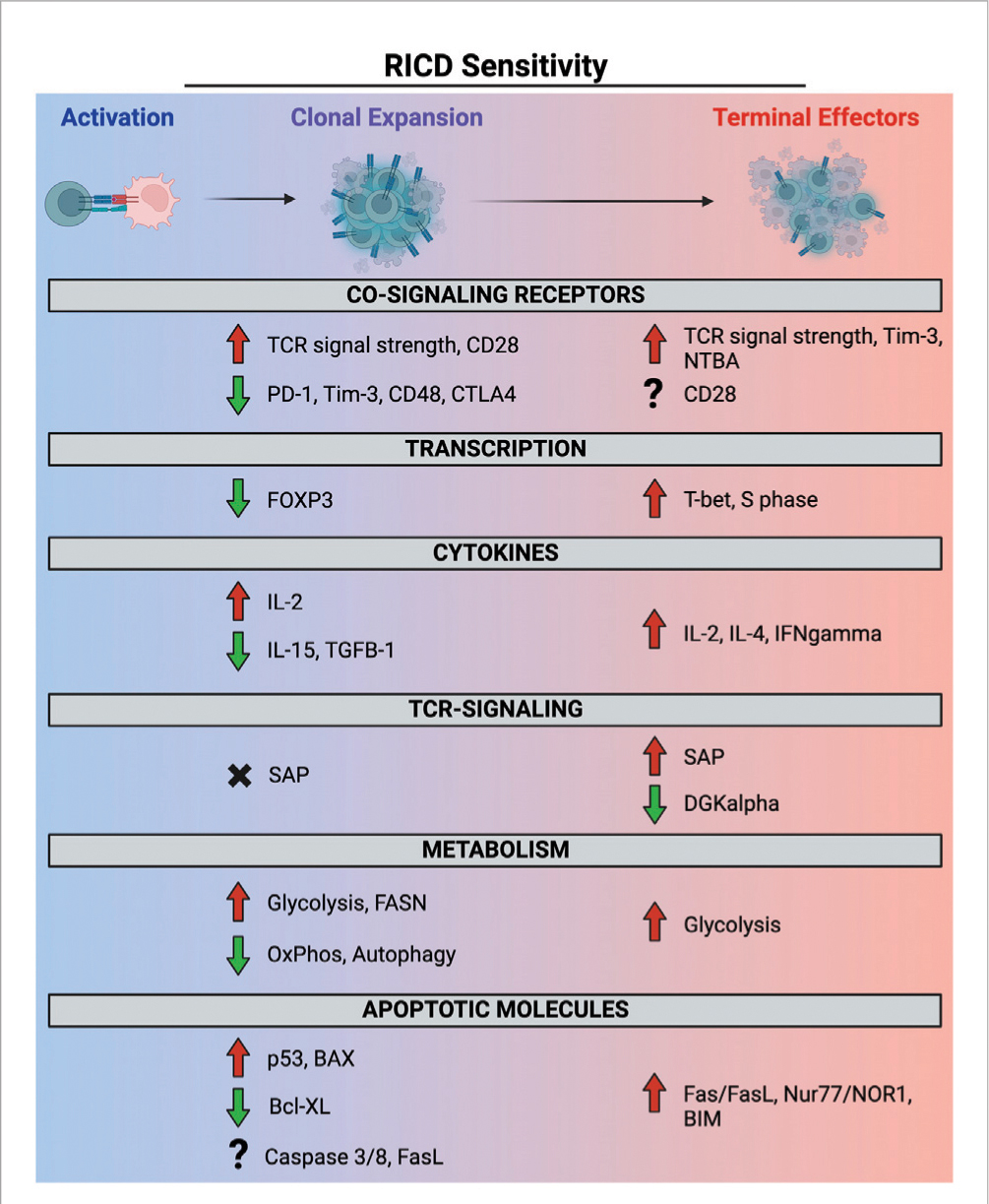
Molecular and metabolic determinants of RICD sensitivity in clonally expanding and terminally differentiated effector T cells. Red and green arrows denote a contribution to RICD susceptibility versus resistance, respectively. SAP expression does not appear to potentiate RICD in early-stage T cells (black X), whereas the precise role of other molecules (e.g., FASL, CD28) at different phases remains poorly defined (question marks).

Repeated TCR engagement in the presence of abundant IL-2 can trigger restimulation-induced cell death (RICD), a propriocidal apoptosis pathway that constrains effector T cell expansion to minimize unintended, non-Ag specific damage to normal tissues. The discovery of this pathway >30 years ago originates with studies in mouse T cell hybridomas (Ashwell et al., 1987) and primary murine T cells (Lenardo, 1991; Russell et al., 1991), whereby TCR engagement was observed to induce cell cycle arrest and/or apoptosis, the latter being eventually dubbed “activation-induced cell death (AICD).” We now appreciate that apoptosis is rarely triggered during the initial Ag encounter by a naïve/resting T cell, making the persistent “AICD” term inaccurate and arguably confusing. Indeed, early expanding human effector T cells (days ~1–4 after Ag encounter) are comparatively resistant to apoptosis induced by TCR restimulation relative to mature effector T cells (days ~7–14) that become “competent to die” via RICD as they reach a terminally differentiated state (Snow et al., 2010; Pohida et al., 2022) (note: the timing here is approximated based on *in vitro* experimentation with human T cells). Moreover, AICD is often cited in textbooks as a major mechanism of self-Ag specific T cell deletion in the periphery. Although autoimmune problems are noted in specific mouse strains and human primary immune disorders linked to genetic defects in intrinsic/extrinsic apoptosis (Bidere et al., 2006), careful examination suggests that RICD is more important for restricting unbridled accumulation of effector T cells responding to infection (Snow et al., 2010). We expound on these points further below as we focus on the molecular mechanisms and functional significance of RICD in T cell homeostasis for the remainder of this review, organized by temporal windows of differential RICD sensitivity during the immune response (Figure 2).

## Setting the limit: RICD in mature effector T cells

Most prior research on RICD has focused on mature effector T cells that proceed past the most proliferative burst of clonal expansion and settle into a terminally differentiated state. Early *in vitro* experiments revealed that although these T cells continue to thrive and divide in the presence of IL-2, TCR restimulation induces a proportion of them to die (Lenardo, 1991; Russell et al., 1991). Shortly after this seminal discovery, the FAS death receptor (i.e., CD95) was implicated (and often equated) with RICD, bolstered by observations of lymphoproliferative disease in both mice and humans that lack a fully functional FAS signaling pathway (Watanabe-Fukunaga et al., 1992; Russell et al., 1993; Gillette-Ferguson and Sidman, 1994; Takahashi et al., 1994; Alderson et al., 1995; Brunner et al., 1995; Dhein et al., 1995; Fisher et al., 1995; Ju et al., 1995). Although the FAS-FASL signaling axis is pertinent, more recent work has illuminated additional pro-apoptotic molecules involved in RICD sensitization and execution (Snow et al., 2010). Collectively, we now appreciate that RICD can be induced in cycling, metabolically active effector T cells that receive a strong TCR signal (self or foreign antigen). In susceptible T cells, robust TCR restimulation induces upregulation of pro-apoptotic molecules like FASL, BIM, and others that participate in RICD execution to varying degrees, depending on CD4/CD8 lineage and differentiation state. RICD is ultimately clonally restricted, providing a mechanism for constraining effector T cell numbers in a highly Ag-specific manner (Lenardo, 1991; Hornung et al., 1997). Nevertheless, even strong polyclonal stimulation (e.g., anti-CD3) cannot induce RICD in all target cells, suggesting mechanisms for tuning RICD sensitivity are in place to ensure enough effector T cells are spared to continue through the CWID-driven contraction phase, with some critical minority persisting into the memory pool. Below we enumerate critical determinants of RICD sensitivity in “late-stage” mature effector T cells (Figure 2).

### Strong proximal TCR signaling

TCR signal transduction is a complex process that requires multiple molecular interactions to stimulate gene transcription, metabolic reprograming, and terminal differentiation. In conventional T cells, the TCR is composed of two transmembrane peptide chains (usually TCRα/β) constructed for unique peptide-MHC (pMHC) sensing, coupled with an assortment of signal transduction molecules collectively known as CD3 (including *δ*, *ε*, *γ*, and *ζ* chains). Expression of co-receptors CD4 or CD8 dictates T cell lineage based on non-Ag-specific binding to class II or class I MHC, respectively. Activation initially occurs when a naïve T cell recognizes unique pMHC complexes (as few as one) on the surface of an antigen-presenting cell (APC), stabilized by CD4/CD8 co-receptor binding and the subsequent formation of an immunological synapse (IS) containing proximal signaling molecules and integrins for sustained T-APC conjugation (Huang et al., 2013). TCR signaling is initiated by LCK-dependent phosphorylation of multiple immunoreceptor tyrosine-based activation motifs (ITAMs) contained in the cytoplasmic tails of associated CD3 molecules. This triggers rapid recruitment of ZAP70, a Syk-family kinase that docks to dually phosphorylated ITAMs via tandem SH2 domains and subsequently phosphorylates numerous proximal signaling molecules in the central IS. Signal amplification via protein phosphorylation cascades and generation of second messengers eventually engages several downstream pathways and key transcription factors (e.g., NF-κB, AP-1, NFAT) that ultimately result in proliferation and acquisition of effector functions (Shah et al., 2021). In naïve T cells, TCR activation is strengthened by costimulation via engagement of CD28 and related molecules. Upon binding to ligands B7–1 and B7–2, LCK-mediated phosphorylation of the CD28 cytoplasmic tail results in PI-3K activation, bolstering proximal TCR signals and inevitably resulting in enhanced cell survival and increased cell metabolism.

Early studies established that a strong TCR signal is required for RICD induction in mature effector T cells via tyrosine phosphorylation on CD3 signaling chains, particularly for CD3ζ which contains 3 distinct ITAMs. The extent of CD3ζ phosphorylation is a semi-quantitative marker of TCR signal strength, with more CD3ζ phosphorylation correlating with increased RICD sensitivity (Combadiere et al., 1996). This harbinger of TCR signal strength reflects a dose-dependent tuning of RICD sensitivity in late-stage terminal effector T cells, with some evidence suggesting ITAMs in additional CD3 signaling chains also play a role in RICD susceptibility (She et al., 1998). Even residues within the TCRβ chain itself can influence qualitative NF-κB signaling output to favor proliferation or apoptosis (Teixeiro et al., 2004).

One might assume that any molecule capable of boosting TCR signal strength would enhance death in this context, but the role of costimulation in RICD remains poorly understood. Although RICD can clearly proceed with TCR re-ligation alone, early work suggested that CD28 costimulation could enhance or antagonize apoptosis in effector T cells depending on concomitant TCR signaling (Collette et al., 1998; Kirchhoff et al., 2000). While CD28 costimulation of naïve T cells is required for naïve T cell activation and subsequent sensitization to RICD (Van Parijs et al., 1996; Boussiotis et al., 1997), re-engagement of CD28 is not necessary to induce RICD in mature Ag-experienced T cells that generally do not require a second CD28 signal for effector function. Nevertheless, work from our group implies that costimulatory or coinhibitory receptors (e.g., SLAM receptor family, TIM-3) may influence RICD by tuning TCR signal strength, discussed in more detail in subsequent sections. Altogether, strong TCR restimulation favors RICD in terminally differentiated effector T cells that exceed a signal threshold for apoptosis commitment (Boehme et al., 1995; Schildberg et al., 2016).

### “Signal 3”: role of cytokines and differentiation state

Cytokines play a key role in the proliferation and differentiation of T cells, constituting an important “3rd signal” for full T cell activation. Both TCR and CD28 signals are required for the transcription of IL-2, considered the most critical “signal 3” for propelling clonal expansion. However, CD4^+^ helper T cell (Th) differentiation states characterized by selective expression of other cytokines also shape T cell responses by regulating cell death sensitivity. In general, Th1 cells are known to be more sensitive to RICD compared to other Th subsets, including Th2, Th17, and Treg cells. Th1 cells (which make IFN-γ) and Th17 cells (which make IL-17) are required for eradicating intracellular and extracellular pathogens, respectively. By contrast, Th2 cells (which make IL-4, IL-5 and IL-13) orchestrate immune responses against helminths and parasites, but are also famously prevalent in many allergic diseases (Zhu, 2018). Differences in the sensitivity of these T cell subsets to RICD have been associated with their respective cytokine profiles and transcription factors; for example, IFN-γ was shown to potentiate RICD via caspase-8 dependent apoptotic process requiring STAT1 (Liu and Janeway, 1990; Refaeli et al., 2002). Given the critical role of the CD95/FAS DR pathway in RICD of CD4^+^ T cells (see below), it is not surprising that differential RICD sensitivity of Th subsets appears to boil down to differences in FAS-FASL expression and function. High levels of IL-2 are known to potently induce the upregulation of DR-ligands like FASL and TNF, which appears more pronounced in Th1 clones (Zheng et al., 1998; Roberts et al., 2003). Simultaneously, IL-2 suppresses transcription of cFLIP, a proteolytically inactive inhibitor of caspases 8 and 10 that blunts FAS-induced apoptosis and is highly expressed in naïve T cells (Refaeli et al., 1998). By contrast, relative RICD resistance in Th17 cells compared to Th1 cells was linked to higher expression of cFLIP coupled with lower FASL induction (Yu et al., 2009; Cencioni et al., 2015). Th2 cells are also more resistant to RICD than their Th1 counterparts, although the molecular underpinnings for this difference remain confusing. Differential FASL induction observed in Th1 vs. Th2 clones offers a clue, although RICD in Th2 cells can proceed through different pathways that are less reliant on caspase activation and more dependent on other molecules like granzyme B (Roberts et al., 2003; Devadas et al., 2006). Enhanced IL-4-driven expression of anti-apoptotic molecules like TID1-S, BCL-2, BCL-xL and cFLIP may also contribute to RICD resistance (Syken et al., 2003; Rautajoki et al., 2007); indeed, different isoforms of cFLIP appear to influence Th differentiation (Kylaniemi et al., 2014). Interestingly, the CD28 antagonist CTLA-4 has also been associated with decreased RICD sensitivity in murine Th effectors, but primarily induces resistance to RICD in Th2 cells upon concomitant CD3/CD28 stimulation due to higher relative expression (Pandiyan et al., 2004). This resistance was associated with decreased Fas and FasL expression, and enhanced Bcl-2 upregulation via PI-3K activity. Finally, regulatory T cells are highly resistant to RICD despite their inherent self-antigen reactivity and dependence on IL-2 for survival and function. The master regulator FOXP3 was shown to suppress the induction of FASL (Weiss et al., 2011), likely through an indirect mechanism involving FOXP3-mediated repression of the adapter SLAM-associated protein (SAP), which is required for optimal RICD induction (see below).

### Cell cycle progression and anabolic metabolism

For naïve T cells, TCR and CD28 signaling synergize to spark clonal expansion by inducing IL-2 transcription and metabolic reprogramming required for rapid T cell proliferation. By promoting cell cycle progression, both are also fundamentally important drivers of RICD sensitization several days after initial Ag encounter. IL-2 plays a fundamental role in immune homeostasis, fostering the survival and proliferation of conventional T cells while concomitantly sustaining sufficient Treg numbers to maintain peripheral tolerance. Whereas IL-2 deprivation is largely synonymous with CWID of conventional effector T cells, IL-2 signaling is required for optimal RICD. Indeed, mice lacking IL-2 or IL-2 receptor exhibit autoimmunity and an excessive buildup of activated T cells (Kneitz et al., 1995; Suzuki et al., 1995; Willerford et al., 1995), reflecting both defective Treg function and RICD. Early experiments established that effector T cells entering or progressing through S phase (i.e., during DNA replication) are specifically sensitive to RICD (Boehme and Lenardo, 1993b). IL-2 signaling is uniquely capable of fueling T cell cycle progression, although other cytokines that signal through the common γ chain can also promote cycling and participate in the induction of FASL and TNF (Zheng et al., 1998; Zhang et al., 2003). Thus IL-2 signaling is intimately involved in feedback regulation of effector T cell numbers at the peak of the response, whereby abundant IL-2 signaling paradoxically limits excessive T cell accumulation by increasing RICD sensitivity via continuous cell cycling. Beyond the induction of pro-apoptotic molecules discussed below, S-phase acceleration may also enhance apoptosis sensitivity through accumulation of DNA damage that exceeds the cell’s repair capacity (Lenardo et al., 1999).

Anabolic metabolism, including glycolysis and fatty acid synthesis, propels the effector T cell response by promoting their proliferation and acquisition of effector functions (e.g., cytokine secretion). Shortly after initial Ag priming, T cells quickly reprogram their metabolic machinery to become much more reliant on glycolysis during clonal expansion, even as they also increase oxidative phosphorylation to meet energetic demands. This dynamic metabolic reprogramming allows for the rapid production of both ATP and macromolecules (derived from glycolytic intermediates) required for T cell proliferation and production of effector molecules like IFN-γ (Chang et al., 2013; Geltink et al., 2018), but also influences apoptosis sensitivity throughout the T cell response (Voss et al., 2017). Recent work from our group suggests that high anabolic metabolism correlates with enhanced RICD sensitivity, which may help to establish an upper limit for expansion of terminally-differentiated, cycling effector T cells that cannot otherwise reprogram to a more metabolically quiescent/stable state required for entry into the memory T cell compartment (e.g., fatty acid oxidation). In human CD8^+^ T cells, we found that RICD sensitivity directly correlated with levels of aerobic glycolysis across multiple individuals. Moreover, inhibition of glycolysis universally reduced RICD, specifically by hampering the induction of FASL (Larsen et al., 2017a). Glycolysis appears to facilitate FASL protein expression by releasing *FASL* mRNA from post-transcriptional repression, perhaps mediated by GAPDH or other glycolytic pathway enzymes with independent moonlighting functions (Larsen et al., 2017a; Sirover, 2018). However, blockade of glycolysis (via 2-deoxyglucose) had no effect on RICD in human CD4^+^ T cells, although inhibition of GAPDH enzymatic activity reduced RICD. Instead, we found that fatty acid synthesis appears to be a major driver of RICD susceptibility in human CD4^+^ effectors by enabling FASL induction (Voss et al., 2019a). Indeed, inhibition of fatty acid synthase (FASN) dramatically protected CD4^+^ effectors from RICD and promoted a Th2-skewed phenotype consistent with reduced RICD sensitivity. More work is required in this space to define how and why pathways of anabolic and catabolic metabolism might influence RICD receptivity in different human effector T cell subsets.

### Death receptors

As mentioned above and reviewed elsewhere, DRs including FAS/CD95 and TNFR1 were implicated in the execution of RICD shortly after the phenomenon was first discovered. Indeed, for many years RICD (a.k.a. AICD) was virtually synonymous with FAS-induced apoptosis, based on compelling evidence in murine CD4^+^ T cell hybridomas and primary CD4^+^ T cells that blockade or genetic mutation of Fas (*lpr* mice) or FasL (*gld* mice) largely abrogated Ag-induced cell suicide (Watanabe-Fukunaga et al., 1992; Russell et al., 1993; Gillette-Ferguson and Sidman, 1994; Takahashi et al., 1994; Alderson et al., 1995; Brunner et al., 1995; Dhein et al., 1995; Ju et al., 1995). Consistent with an apoptosis defect, *lpr* and *gld* mice both exhibit signs of lymphoproliferation (including enlarged lymph nodes and spleen, and accumulation of atypical CD4^−^CD8^−^ “double negative” T cells) and mild autoimmune manifestations resembling lupus-like disease. The discovery of humans with a similar lymphoproliferative phenotype and harboring heterozygous loss-of-function *FAS* mutations, dubbed Autoimmune Lymphoproliferative Syndrome (ALPS), further solidified FAS-induced apoptosis as a critical player in maintaining lymphocyte homeostasis (Sneller et al., 1992; Fisher et al., 1995; Rieux-Laucat et al., 1995). Autoantibody-driven autoimmune cytopenias and B cell lymphomas are also noted in these patients with variable penetrance (Oliveira et al., 2010; Consonni et al., 2022). Subcategories of ALPS were subsequently recognized, linked to somatic FAS variants or LOF/null mutations in other FAS pathway genes (e.g., FASL, caspase 10) (Wu et al., 1996; Wang et al., 1999; Holzelova et al., 2004). Patients carrying deleterious variants in FADD and caspase 8 exhibit ALPS-like lymphoproliferation and broader, more severe phenotypes consistent with immunodeficiency, given the pleiotropic roles of both proteins in other signaling pathways (Chun et al., 2002; Bolze et al., 2010; Savic et al., 2015; Kohn et al., 2020). ALPS-like phenotypes linked to diminished RICD sensitivity can even extend to pathogenic variants in genes outside the FAS/CD95 signaling pathway, including KRAS/NRAS (RAS-associated leukoproliferative disease) and STAT5 (Table 1) (Snow et al., 2008; Calvo et al., 2015; Majri et al., 2018).

Continued research over the years has confirmed that FAS-FASL interactions play a major role in mediating RICD in CD4^+^ T cells, depending on their differentiation state (see above). The contribution of FAS-induced apoptosis to CD4^+^ T cell RICD is largely determined by (a) the extent of IL-2-dependent FASL upregulation, and (b) proper localization of FAS trimers to lipid raft microdomains, and (c) the efficiency of FAS-induced DISC formation for downstream caspase activation, which may or may not require amplification through BID cleavage and intrinsic, mitochondria-dependent activation of caspase 9 (Barnhart et al., 2003; Schmitz et al., 2003; Ramaswamy et al., 2011). Furthermore, TCR restimulation itself determines if a T cell is receptive to FAS-induced apoptosis, explaining why RICD is highly Ag-specific even in the presence of activated, bystander FAS/CD95+ T cell clones bearing different TCRs (Hornung et al., 1997). Again, sensitivity is ultimately adjudicated by the strength and specificity of the TCR restimulation signal—only co-engagement of TCR and FAS/CD95 enables FASL-FAS dependent apoptosis during under physiologic conditions (Zheng et al., 2017).

In CD8^+^ T cells, FAS plays a minor role in RICD susceptibility—TCR-induced apoptosis is largely intact in CD8^+^ effector T cells derived from *lpr/gld* mice or ALPS patients (Zimmermann et al., 1996; Snow et al., 2008). In fact, *in vivo* Ag-driven T cell expansion and contraction is relatively normal in conditional knockout mice where FAS is selectively eliminated in T cells, despite the persistence of chronically stimulated DNTs and autoantibody production (Stranges et al., 2007). Some studies suggest that TNF signaling can promote RICD (Zheng et al., 1995), while others suggest its role is limited (Gorak-Stolinska et al., 2001). TNFR1 is a true DR that can participate in CD8^+^ T cell RICD after TNF engagement, but the precise role of TNFR1 in T cell apoptosis is more opaque. This relates to the complicated and dynamic composition of TNFR1-induced DISC complexes, which can also promote NF-κB activation and/or necroptosis (a distinct form of programmed cell death) in partnership with TRAF2 or RIPK1, respectively (Zheng et al., 2017). When naïve CD8^+^ T cells fail to procure adequate help from CD4^+^ T cells during priming, the resulting effectors are also susceptible to TRAIL-mediated apoptosis signaled through death receptors 4 or 5 (DR4/DR5) (Janssen et al., 2005). Unlike *lpr* or *gld* mice, however, mice lacking TNF, TRAIL, or their respective DRs show no appreciable defects in lymphocyte homeostasis, suggesting these FAS-independent DR pathways play a minimal role in RICD. Moreover, sophisticated mouse models in which Fas or FasL is only removed from T cells, B cells or dendritic cells all share lymphoproliferation and systemic autoimmune features (Stranges et al., 2007; Mabrouk et al., 2008), underscoring the concept that FAS/CD95 safeguards against autoimmunity by deleting chronically activated, self-reactive lymphocytes and clearing excess APCs. Even this idea may require revision (see below). As explained below, we now understand that additional pro-apoptotic molecules participate in RICD of normal non-self reactive T cells, especially in the CD8^+^ compartment.

### Additional pro-apoptotic molecules

Loss of IL-2 signaling causes the dramatic upregulation of BIM, a “BH3-only” pro-apoptotic member of the BCL-2 family required for CWID. Intriguingly, several lines of evidence suggest BIM is also involved in RICD, particularly in CD8^+^ T cells (Grayson et al., 2006). First, we and others found that BIM is efficiently upregulated in human effector T cells after TCR restimulation, even in the presence of high IL-2 (Sandalova et al., 2004; Snow et al., 2008). Second, siRNA-mediated silencing of BIM upregulation partially rescued human effector T cells from RICD, with more pronounced resistance observed in CD8^+^ T cells (Snow et al., 2008). Third, acute overaccumulation of CD8^+^ T cells was observed in Bim−/− mice infected with viruses (e.g., LCMV, HSV-1), well before viral Ag is cleared and CWID takes hold (Pellegrini et al., 2003; Grayson et al., 2006). Finally, effector CD8^+^ T cells derived from ALPS-Ia patients (harboring germline FAS mutations) express more BIM than normal donor controls, showing comparable levels of death after polyclonal TCR restimulation *in vitro* (Snow et al., 2008). These studies all implied that BIM plays a prominent role in CD8^+^ T cells, and can even compensate for compromised FAS signaling if necessary to maintain T cell homeostasis. The derivation of Bim-deficient *lpr* mice in 2008 cemented this conclusion: compared with single knockouts alone, loss of both Bim and Fas resulted in extraordinary lymphoproliferation with massive lymphadenopathy, splenomegaly and DNT accumulation, coupled with early-onset, fatal lupus-like autoimmunity (Hughes et al., 2008; Hutcheson et al., 2008; Weant et al., 2008). Uncontrolled T cell expansion was observed in response to several viral challenges. Similar phenotypes were observed in Bcl2-transgenic *lpr* mice, in which Bcl2 overexpression counteracts the critical pro-apoptotic function of Bim (Reap et al., 1995). Collectively, these studies firmly establish BIM and FAS/CD95 as the primary gatekeepers of T cell homeostasis, including scenarios where acute or persistent Ag remains abundant and RICD must be engaged to check unbridled effector T cell expansion.

Additional pro-apoptotic molecules have been implicated as minor players in RICD execution. The perforin/granzyme pathway that anchors effector CD8^+^ T cell cytotoxic function can also contribute to RICD, compensating for defective FAS-mediated apoptosis in ALPS Ia patents (Mateo et al., 2007). Two steroid nuclear receptors, NUR77 and NOR1, also participate as functionally redundant pro-apoptotic proteins involved in clonal deletion of thymocytes and mature effector T cells (Cheng et al., 1997). Both are strongly induced after TCR stimulation and subsequently phosphorylated by the ERK-dependent ribosomal S6 kinase (RSK), which triggers nuclear export and translocation to the mitochondria (van den Brink et al., 1999; Wang et al., 2009). Phospho-NUR77/NOR1 can then induce the intrinsic apoptosis pathway via mitochondrial depolarization. As explained below, we discovered that this process requires a strong TCR signal enabled by SAP-dependent inhibition of diacylglycerol kinase alpha (DGKα) (Ruffo et al., 2016). Overall, the relative contribution of various pro-apoptotic molecules to RICD sensitivity remains difficult to define and likely varies depending on T cell lineage, differentiation state, and context (e.g., nature of antigen/infection, etc.). Nevertheless, the involvement of multiple pro-apoptotic molecules, often with functional redundancy or compensatory capability, underscores the essential role of TCR-induced apoptosis in eliminating self-reactive T cells and more importantly, taming clonal expansion to ensure immune homeostasis. As we discuss below, defective RICD-mediated control of effector T cell accumulation in patients with X-linked lymphoproliferative disease 1 (XLP-1) can have fatal consequences (Table 1).

### SAP: reaching the threshold for RICD

XLP-1 is a rare immune disorder associated with various immune defects in boys, including impaired cytotoxic T lymphocyte (CTL) function, hypo-/dysgammaglobulienemia, and loss of natural killer T (NKT) cells (Panchal et al., 2018), with congruent phenotypes recapitulated in Sap-deficient mice (Czar et al., 2001). However, life-threatening disease in patients is rarely observed until infection with Epstein-Barr virus (EBV), which sparks uncontrolled accumulation of CD8^+^ effector T cells that infiltrate and damage multiple organs/tissues (Bar et al., 1974). This immunopathology can manifest as fulminant infectious mononucleosis (FIM), hemophagocytic lymphohistiocytosis (HLH), macrophage activation syndrome (MAS), and/or vasculitis (Purtilo et al., 1975; Seemayer et al., 1995). XLP-1 is caused by null/LOF mutations in the X-linked *SH2D1A* gene encoding SAP, a small adaptor protein comprised of a single SH2 domain (Tangye, 2014). SAP partners with a variety of signal lymphocyte activation molecule family receptors (SLAM-Rs), which provide crucial costimulatory functions in lymphocytes. SLAM-R engagement (often through homotypic interaction) invites SAP to dock with phosphorylated immunoreceptor switch motifs (ITSM) in the receptor cytoplasmic tail, often by displacing pre-associated inhibitory phosphatases like SHP-1/2. SAP further enables recruitment of Src family kinases (FYN, LCK) to propagate downstream signals important for Th2 differentiation, B cell help and cytotoxic function. The complicated molecular mechanisms surrounding SAP-SLAM-R signaling are reviewed in detail elsewhere (Cannons et al., 2011; Dragovich and Mor, 2018; Acharya et al., 2020).

Early studies in Sap-deficient mice also noted aggressive CD8 T cell expansion and immunopathology upon viral infection, mimicking EBV-infection in XLP-1. But how is SAP deficiency linked to this acute and potentially fatal flaw in Ag-directed T cell homeostasis, which appears distinct from the smoldering lymphoproliferation and autoimmunity observed in ALPS? Prior work has firmly established that T cells derived from XLP-1 patients are profoundly resistant to RICD (Snow et al., 2009), with a similar defect observed in SAP knockout mice (Chen et al., 2007). SAP expression is relatively low in naïve T cells, but steadily increases during Ag-driven clonal expansion (Shinozaki et al., 2002; Mehrle et al., 2005). Upon mild to moderate TCR restimulation of effector T cells, SAP associates with a specific SLAM-R known as NK, T, and B cell antigen (NTB-A) to effectively boost TCR signal strength above a threshold required for the optimal induction of several downstream pro-apoptotic proteins, including FASL, BIM, NUR77, and NOR1. Knockdown of SAP or NTB-A recapitulates the RICD defect in effector T cells from normal human donors, with more profound impairment noted in CD8^+^ T cells. Stronger TCR restimulation abrogates the requirement for SAP-NTB-A costimulation, suggesting that relative RICD resistance could correlate with specific Ags and/or TCR affinity of specific clones. Subsequent work from our group illuminated how SAP augments TCR signal strength by (a) displacing SHP-1 and recruiting LCK to the cytoplasmic tail of NTB-A, converting it from an inhibitory to activating receptor, and (b) inhibiting DGKα to conserve sufficient diacylglycerol (DAG) levels for robust proximal TCR signaling (Snow et al., 2009; Baldanzi et al., 2011; Katz et al., 2014; Ruffo et al., 2016). DGKα is responsible for converting DAG to phosphatidic acid (Wattenberg and Raben, 2007), and its enzymatic activity is elevated in the absence of SAP (Baldanzi et al., 2011). Remarkably, pharmacological inhibition of DGKα partially restored RICD sensitivity in XLP-1 patient effector T cells, and markedly reduced CD8^+^ T cell accumulation and overt immunopathology in Sap-deficient mice infected with LCMV (Ruffo et al., 2016). Improved RICD in this context was attributed to partial rescue of NUR77 and NOR1 induction following TCR restimulation, whereas FASL and BIM levels remained low with DGKα inhibition. These results imply that SAP potentiates RICD through both DGKα-dependent and independent mechanisms. New work reveals that SAP appears to inhibit DGKα through a multiprotein signalosome incorporating NCK-1, CDC42 and WASp, tuning both IL-2 secretion and RICD susceptibility through distinct SAP-dependent pathways that require further characterization (Velnati et al., 2023). Nonetheless, our study provided an important proof-of-concept that pharmacological inhibition of a single enzyme can correct deranged immune homeostasis in human patients specifically by enhancing RICD sensitivity, unveiling a novel therapeutic approach for XLP-1 (Velnati et al., 2019). Although original DGKα inhibitors have demonstrated off-target effects and poor pharmacokinetics, more recent studies have identified ritanserin (a 5-HT receptor antagonist) and AMB639752 (highly specific DGKα inhibitor) as promising candidates capable of restoring RICD sensitivity in SAP-deficient T cells (Velnati et al., 2019).

We posit that XLP-1 represents the best known example of a potentially fatal immune disease directly linked to an RICD defect, triggered by a single, B cell tropic pathogen: EBV. Myriad studies have established the unique role of SLAM-R signaling specifically in T cell interactions with B cells, in contrast to other APCs (Hislop et al., 2010). SAP and SLAM-Rs like NTB-A (i.e., Ly108 in mice) are crucial for T:B cell conjugation, germinal center formation, and CD8-mediated killing of infected B cells (Schwartzberg et al., 2009). Upon EBV infection in XLP-1 patients, suboptimal T:B cell interactions contribute to poor cytotoxic elimination of EBV-infected B cells, heightening the risk of B cell lymphomagenesis. Abundant EBV antigen contributes to repeated, weak restimulations of CD8^+^ effector T cells that cannot achieve the signal threshold required for RICD induction in the absence of SAP. Hence, CD8^+^ effector T cells persist and accumulate uncontrollably in this context, manifesting in FIM and related immunopathologies that wreak severe damage in multiple tissues (Snow et al., 2010). Given the broader role SAP plays in boosting TCR restimulation and enhancing the upregulation of multiple pro-apoptotic molecules, we now comprehend the fatal flaw of true RICD resistance in XLP-1 patients, in contrast to genetic defects in a single, downstream apoptotic pathway (e.g., Fas-induced defects in ALPS). Further studies are needed to determine whether inhibition of modulatory enzymes like DGKα or SHP-1/2 phosphatases can be clinically effective in taming CD8^+^ lymphoproliferation in XLP-1. A recent study beautifully showed that inhibition of SHP-2 could correct several immune defects caused by SAP-deficiency in mice, including restoration of RICD (Panchal et al., 2022).

Fascinatingly, it appears dysregulation of RICD and the SAP-NTB-A axis extends beyond XLP-1; a recent study of tuberculosis patients found that patient-derived T cells that respond poorly to *M. tuberculosis* antigens are resistant to RICD. In this scenario, preferential SAP-directed recruitment of FYN to NTB-A skews CD4^+^ T cells to a Th2 phenotype and fails to effectively induce death upon TCR restimulation (Hernandez Del Pino et al., 2017). There are also likely additional inborn errors of immunity that feature abnormal RICD sensitivity as a driver of pathogenesis, independently of abnormal SAP/NTB-A signaling. A recent study described an ALPS-like patient harboring a dominant negative *STAT5B* mutation that reduced IL-2R-induced transcriptional activity. The patient displayed an overaccumulation of circulating CD4^+^ effector memory (EM) T cells, which are normally highly sensitive to TCR-induced apoptosis (Ramaswamy et al., 2011; Hernandez Del Pino et al., 2017; Majri et al., 2018). This phenomenon was phenocopied in Stat5b-deficient mice. Moreover, direct inhibition of IL-2R, JAK3 or STAT5B conferred marked RICD resistance in EM cells isolated from healthy donors, confirming the essential role of IL-2 signaling in RICD sensitization.

## Relative RICD resistance during clonal expansion

Although we now understand several determinants of RICD susceptibility in terminally differentiated effectors, recently activated T cells apparently do not follow the same paradigm. During early stages of clonal expansion, when IL-2 and antigen are abundant, T cells are relatively resistant to RICD. The transition from RICD resistance to sensitivity in mature effector T cells is physiologically advantageous for building an adequate pool of effectors to combat infection without exceeding a number that could be damaging to the host. The transcriptome and proteome of clonally expanding, differentiating T cells certainly diverges substantially from terminally differentiated cells, but how these variations afford and dictate apoptosis sensitivity remains poorly understood. Moreover, even similar expression of classic pro-apoptotic molecules in both early and late-stage effectors makes RICD resistance in the former even more perplexing. For example, early reports showed differences in classic mediators of TCR-induced apoptosis such as FAS/CD95. One day after activation, T cells exhibited high BCL-xL expression and low FAS-mediated apoptosis sensitivity. This phenotype changed by day 6 post-stimulation, demonstrated by greater FAS-induced cell death (Peter et al., 1997). Subsequent reports have revealed non-apoptotic, costimulatory functions for FAS-FASL signaling, which can influence inflammation, effector T cell fates and memory formation (Yi et al., 2018). Whereas apoptosis requires palmitoylation of FAS trimers for lipid raft localization and enhanced multimerization (Muppidi and Siegel, 2004), FAS can also signal to NF-κB, ERK, and AKT outside of lipid rafts (Yi et al., 2018). In this manner, non-apoptotic FAS signaling has been shown to prevent lymphoproliferation and autoimmunity, and promote proliferation and “precocious differentiation” of naïve T cells, with implications for adoptive T cell therapies (Alderson et al., 1993; Cruz et al., 2016; Klebanoff et al., 2016). Indeed, other pro-apoptotic molecules like caspase 8 and FADD exhibit pleiotropic functions in TCR and non-apoptotic DR signaling, further complicating any model of temporally controlled RICD sensitization. Over the last decade, however, research from our group and others has begun to shed light on the molecular and metabolic underpinnings of RICD resistance in early expanding T cells (Figure 2). In fact, differences in apoptotic sensitivity amongst T cell subpopulations provided early clues about specific proteins and signaling pathways that help to ensure cell survival.

### FOXP3 modulates RICD sensitivity in both regulatory and conventional T cells

CD4^+^ Tregs are a unique class of T cells required to enforce peripheral tolerance and maintain immune homeostasis (Shevyrev and Tereshchenko, 2019). The fate and repressive function of Tregs is ultimately governed by the master regulator transcription factor FOXP3, in partnership with numerous accessory transcription factors (e.g., Helios) that help to explain Treg heterogeneity (Trujillo-Ochoa et al., 2023). Numerous studies have characterized several distinct mechanisms by which Tregs can suppress conventional T cell (Tcon) responses, reviewed in detail elsewhere. Given that Tregs are inherently self-reactive and require IL-2 for development and maintenance, how is this population spared from RICD-mediated elimination in the face of repeated TCR restimulations in IL-2, self-antigen rich environments? Disparity in programmed cell death between Tregs versus Tcons was first revealed as differences between TCR- and FAS-induced apoptosis. Although activated Tregs express FAS/CD95, FOXP3 is required to blunt RICD sensitivity by suppressing FASL induction upon TCR restimulation (Fritzsching et al., 2005; Weiss et al., 2011). More recently, we showed that FOXP3 itself suppresses SAP expression in Tregs via transcriptional repression, explaining why TCR restimulation cannot induce enough FASL expression to trigger death (Katz et al., 2018). Interestingly, TGF-β1 also mediates apoptotic resistance of Tregs *in vivo* in late stages of infection, possibly by upregulating cFLIP (Bhaskaran et al., 2016).

Intriguingly, FOXP3 is transiently upregulated in recently activated conventional human (but not murine) T cells. Although this short-lived FOXP3 expression is not sufficient to endow Tcons with suppressive function, conflicting reports suggest it may regulate Tcon proliferation and cytokine production (Allan et al., 2007; Wang et al., 2007; Cavatorta et al., 2012; McMurchy et al., 2013). Because FOXP3 is expressed in activated expanding T cons when antigen and IL-2 are abundant, we reasoned that transient FOXP3 upregulation may protect activated Tcons from premature RICD by suppressing SAP expression, mirroring its function in Tregs. We showed that siRNA-mediated knockdown of FOXP3 significantly enhanced RICD sensitivity during *in vitro* clonal expansion of human T cells, a finding not conferred in late-stage, terminally differentiated T cells (Voss et al., 2019b). However, FOXP3 silencing did not impact SAP expression, and SAP and NTB-A were not required for residual RICD at this early stage. While SAP re-expression was sufficient to sensitize FOXP3+ Treg cells to RICD (Katz et al., 2018), FOXP3 expression in conventional T cells is naturally low and may require other transcription factors to regulate RICD in early stage Tcons, which is entirely reliant on *de novo* transcription.

Further experiments revealed that CD48, a SLAM family-related receptor and transcriptional target of FOXP3, confers RICD resistance in early-stage effectors by sustaining basal autophagy and suppressing p53-mediated apoptosis upon TCR restimulation. TCR-induced selective autophagy and macroautophagy have been shown to help to regulate NF-κB activation and enable clonal expansion, respectively, in part by promoting T cell survival (Pua et al., 2007; Paul et al., 2012). Although the connection between macroautophagy and p53 regulation is well recognized in tumor cells (Rahman et al., 2022), its unforeseen involvement in governing RICD resistance highlights a broader network of pathways required for managing early-stage T cell durability, including cellular metabolism.

### Early metabolic reprogramming influences RICD sensitivity

Immunometabolism remains a growing, exciting field that showcases how lymphocytes rapidly adapt to their environment and perform effector functions via dynamic metabolic reprogramming. In T cells, a dramatic shift from oxidative phosphorylation to predominantly anabolic, glycolytic metabolism after T cell activation helps to enable redox balancing, macromolecule biosynthesis, and effector cytokine secretion (Buck et al., 2015). This metabolic shift requires extensive transcriptional reprogramming (e.g., induction of c-Myc, ERRα, HIF-1alpha, and mTOR), growth factors (e.g., IL-2) and heightened nutrient utilization (e.g., glucose, glutamine, amino acids, and lipids) for efficient effector cell expansion and differentiation. Unsurprisingly, metabolic status also influences apoptotic sensitivity over the course of a T cell response (Voss et al., 2017). In late-stage human CD8^+^ effector T cells, we found that RICD sensitivity directly correlates with aerobic glycolysis, which specifically facilitates robust FASL induction upon TCR restimulation (Larsen et al., 2017a). Dampening glycolytic flux via pharmacological inhibition (e.g., 2-deoxyglucose) or altered fuel utilization (e.g., galactose versus glucose) significantly reduced RICD sensitivity. This was the first study to directly connect RICD susceptibility to anabolic metabolism, and suggested that T cells destined for longer-term survival in the memory pool must switch back to a more metabolically quiescent state to avoid RICD. This helps to clarify the role of specific cytokines in this process. For example, IL-2 was found to increase active caspase-3 levels, whereas its memory-promoting cousin cytokine IL-15 conferred RICD resistance by dampening caspase-3 by S-nitrosylation and glutathionylation (Saligrama et al., 2014). Further studies revealed that IL-15 increased ROS, oxygen consumption rate, and electron transport chain activity, effectively reorienting T cells to oxidative metabolism (Secinaro et al., 2018; Secinaro et al., 2019). Comparing these different metabolic states dictated by relative cytokine levels revealed that proper localization and cleavage of caspase-3 is more pronounced in glycolytically-dominant T cells, inferring greater RICD sensitivity.

These studies implied that metabolic programming can play an important role in regulating cell death in clonally-expanding and terminally-differentiated effector T cells. Indeed, other cellular metabolic programs have now been linked to RICD in CD4^+^ T cells. In performing a limited screen of pharmacologic inhibitors, we found that blockade of fatty acid synthesis, but not fatty acid oxidation, decreased TCR-induced apoptosis in mature CD4^+^ and CD8^+^ effector T cells (Voss et al., 2019a). By measuring oxygen consumption and extracellular acidification rates, we found that inhibition of fatty acid synthase (FASN) fundamentally altered cellular bioenergetics, reducing basal and maximal mitochondrial respiration, glycolytic flux, and ATP production. Yet in contrast to CD8^+^ T cells, disruption of glycolysis itself did not decrease RICD sensitivity in CD4^+^ effectors, although direct GAPDH inhibition did. FASN inhibition reduced TCR-mediated upregulation of multiple pro-apoptotic molecules including NUR77, BIM, and most importantly FASL, and skewed CD4 effector T cells to a more Th2-like phenotype associated with decreased RICD sensitivity (see above). Further studies are warranted in early expanding T cells, but these results further underscore the impact of the switch to heightened anabolic metabolism on T cell survival and differentiation via RICD regulation.

TCR-stimulation induces other catabolic adaptations, one of which is closely linked to apoptosis and the highly-conserved, lysosomal degradation process known as macroautophagy (hereafter refered to as autophagy). Autophagy is an autonomous, homeostatic process used in nearly all eukaryotic cells that recycles cytoplasmic material to generate metabolites and ATP in response to growth factor/nutrient starvation and/or physiological stress, enabling cell survival (Merkley et al., 2018). Indeed, T cells also utilize this pathway during thymocyte development, activation, and Th differentiation in response to certain cytokines. In fact, autophagy is required for an effective T cell response; autophagy-deficient T cells have dysregulated ATP and substrate degradation which leads to imbalances in glycolysis, oxidative phosphorylation, and fatty acid oxidation (Dowling and Macian, 2018). Autophagy is inherently connected to immunometabolism via numerous nutrient sensors, transporters, and critical signal regulators such as mTOR and AMPK. Our lab and others have demonstrated the how apoptosis sensitivity can be tuned via autophagy in human T cells. CD8^+^ effectors derived from central memory (CM) T cells exhibit sustained autophagy that affords CWID resistance compared to effector memory (EM)-derived counterparts (Larsen et al., 2017b). Knockout of Beclin-1, an autophagy related gene, induces programmed cell death upon TCR stimulation by upregulating BIM and caspases 8 and 3 (Kovacs et al., 2012). In Jurkat and late-stage effector T cells, TCR-induced autophagy must be restrained via protein kinase A (PKA)-mediated suppression of AMPK for optimal RICD, via the accumulation of damaged mitochondria that facilitate apoptosis through enhanced cytochrome c release (Corrado et al., 2016). In early activated T cells, we have already discussed RICD resistance imparted by FOXP3-induced CD48-dependent autophagy (see above). More work is clearly needed to understand how relative reliance on anabolic and catabolic metabolism programs ultimately tune programmed cell death sensitivity in T cells during clonal expansion.

### Tuning TCR signal strength: costimulatory and coinhibitory receptors

Amongst the many intracellular changes occurring after initial T cell activation, surface molecules are in constant flux, influencing cell:cell interactions and adjusting TCR activation signals through both co-stimulation and co-inhibition. Because RICD requires a strong TCR signal, it seems logical that surface receptors that directly and indirectly fine-tune signaling cascades downstream of the TCR may influence RICD sensitivity. Indeed, NTB-A provides a poignant example in mature effectors, with SAP dictating its stimulatory versus inhibitory role in RICD (Snow et al., 2009). One relevant model that demonstrates the importance of co-signaling proteins is CD8^+^ T cell exhaustion, in which various co-inhibitory molecules serve as “checkpoints” to attenuate TCR signaling in the context of chronic antigen restimulation (McLane et al., 2019). Under normal circumstances, acute infection rapidly generates a cytotoxic effector T cell pool that eventually dwindles to a small percentage of memory cells after antigen clearance. Failure to clear antigen and appropriately enter the contraction phase in conditions of prolonged antigen exposure, such as cancer or chronic viral infection, results in exhaustion (McLane et al., 2019). Exhausted T cells are transcriptionally and epigenetically reprogrammed in the face of chronic antigen stimulation to induce a state of diminished effector function, metabolic dysregulation, and most notably, TCR hyporesponsiveness due to elevated co-inhibitory receptor expression. Co-inhibitory receptors such as PD-1, TIM-3, LAG-3, CTLA-4, and TIGIT impart negative signals during TCR engagement that blunt activation, slow cell cycle progression and metabolic activity, and affect memory T cell generation. By precluding and/or reversing exhaustion to rejuventate CD8 effector T cell function, “checkpoint blockade” molecules targeting PD-1/PD-L1 and CTLA-4 have revolutionized immunotherapy for a variety of aggressive cancers (Sharma et al., 2023). Given the profound impact of coinhibitory receptors in exhausted T cells, we and others have hypothesized that early upregulation of co-inhibitory receptors ultimately helps to spare some T cells from RICD, defining exhaustion as a necessary mechanism for surviving a hostile environment of overwhelming antigen abundance such as the tumor microenvironment.

Previous work has discovered a role for both coinhibitory and costimulatory molecules in T cell apoptosis, albeit with confusing results. Costimulatory molecules CD28, OX40, and CD27 have classically been associated with apoptotic resistance in the event of T cell (re)stimulation (Chen and Flies, 2013; Blake et al., 2022). However, subsequent data from our lab and others suggest CD28 engagement does not have an appreciable effect on RICD, and might serve to boost RICD sensitivity slightly in expanding CD4^+^ T cells. Microarray studies identified the anti-apoptotic protein BCL-xL to be upregulated by TIGIT stimulation (Joller et al., 2011), even though agonistic anti-TIGIT antibodies can induce cell death in certain memory T cells in the context of transplant rejection (Sun et al., 2021). LAG-3 and PD-1 may also regulate apoptosis in the setting of autoimmune disease, infection, or cancer (Ostrand-Rosenberg et al., 2014; Lou et al., 2020; Jones et al., 2022)—indeed, PD-1 was initially assigned a pro-apoptotic role in early studies (Ishida et al., 1992). CTLA-4, another coinhibitory protein that competes with CD28 for binding to B7–1 and 2, was implicated in TCR-mediated apoptosis resistance by reducing FASL expression in T cell hybridomas, a function mirrored in Th2 cells via PI-3K-dependent phosphorylation of FKHRL1 and BCL-2 upregulation (Pandiyan et al., 2004; da Rocha Dias and Rudd, 2001).

However, most studies have not examined co-inhibitory receptor function during clonal expansion. The expression of several co-inhibitors is significantly elevated in recently activated T cells (e.g., PD-1, TIM-3), long before exhaustion can be established. Recently, our lab demonstrated an RICD-protective role for TIM-3 during clonal expansion; a temporal effect that reversed in mature effector T cells that may be more prone to severe, terminal exhaustion (Lake et al., 2021). TIM-3 partners with surface ligand carcinoembryonic antigen-related cell adhesion molecule 1 (CEACAM1) in early expanding CD8^+^ T cells to help prevent premature RICD through a still unknown mechanism. In contrast, TIM-3 remained largely intracellular and potentiated RICD sensitivity in late-stage terminally differentiated effectors with diminished expression of CEACAM-1. These findings may have implications in explaining T cell dyscrasias like subcutaneous panniculitis-like T cell lymphoma (SPTCL), with >60% cases arising in patients with germline TIM-3 mutations that likely disrupt CEACAM-1 binding and surface localization (Gayden et al., 2018). Furthermore, ongoing work in our lab has also revealed a potential role for PD-1 in promoting T cell survival during clonal expansion. PD-1 is rapidly induced in expanding CD4^+^ and CD8^+^ human T cells *in vitro*, but expression quickly drops by 7 days post-stimulation. We found that PD-L1 engagement of PD-1 in the immunological synapse markedly reduces apoptosis upon TCR restimulation, dampening proximal TCR signaling and altering the relative expression of both pro- and anti-apoptotic molecules downstream (manuscript in preparation). These tantalizing initial insights demand more research into if and how other cosignaling molecules preserve immune homeostasis by adjusting RICD susceptibility over time, in both normal and disease settings.

## Future directions

Despite the progress accomplished in recent years, further research is required to improve our understanding of cellular and biochemical mechanisms that govern T cell apoptosis sensitivity across the entire life span of a T cell clone. Careful examination of RICD in both clonally expanding and terminally differentiated effector T cells has already highlighted complex molecular networks that tune and refine death sensitivity. These findings can inform therapeutic interventions that can manipulate the magnitude and potency of an effector T cell response based on relative RICD sensitivity; the potential utility of DGKα or SHP-2 inhibitors in XLP-1 presents a notable example (Ruffo et al., 2016; Panchal et al., 2022). Moreover, forced RICD via antigen overload represents a potentially attractive strategy for ameliorating autoimmune disease via clonal deletion of self-reactive T cells, with proof-of-concept studies already achieved in murine models (e.g., experimental autoimmune encephalitis) (Critchfield et al., 1994; Li et al., 2023). Before relevant clinical treatments can come to fruition, future research on RICD must continue to dissect differences in surface receptor expression, intracellular signaling networks, metabolic programming, and downstream gene transcription in effector T cells derived from naïve, effector, and memory cell subsets. In turn, these efforts will hopefully clarify and pinpoint key molecular targets for boosting RICD sensitivity to reduce lymphoproliferation and autoimmunity, or attenuating RICD when more effector T cells are required to combat infection or cancer (including adoptive T cell therapies). Below we briefly consider areas for further investigation.

### Co-signaling receptors

Striking the right balance of activating and inhibitory signals to preserve robust T cell responses without altering RICD sensitivity remains a formidable challenge in cancer immunotherapy, including adoptive and chimeric antigen receptor (CAR) T cell therapies (Chhabra, 2011; Huan et al., 2022). Given our findings on TIM-3 and PD-1, it would be worthwhile to consider how other co-inhibitory proteins regulate T cell equilibrium and RICD. In recent years, checkpoint inhibition has emerged as the dominant strategy for reinvigorating T cell responses to help fight difficult cancers. Antibodies against CTLA-4 and PD-1 are now approved for use in at least 17 different malignancies (Johnson et al., 2022), with combination therapies arising as a potent approach. Other T cell co-inhibitory molecules are being investigated for therapeutic inhibition as well, including LAG-3, TIGIT, VISTA, CD160, and CD244 (Baumeister et al., 2016). Despite exciting advancements, checkpoint inhibitors are not effective in all patients. Moreover, clinicians have encountered a variety of immune-related adverse events (IRAEs) in response to these drugs, prompting a newfound appreciation for systemic effects unrelated to cancer (Bagchi et al., 2021; Kichloo et al., 2021; Conroy and Naidoo, 2022; Johnson et al., 2022). Could any of these shortcomings relate to effects on tumor non-specific T cell responses, including abnormal apoptosis? For example, Tregs found in patients with checkpoint-induced IRAEs exhibit a pro-inflammatory, apoptotic gene signature with altered metabolism (Grigoriou et al., 2021). More research is required to understand how co-inhibitory molecules influence conventional and regulatory T cell dynamics in healthy and cancer patients, with RICD sensitivity as an important rheostat. Affected pathways often intersect with those induced by various co-stimulatory molecules such as CD28, ICOS, OX40, 41BB, and CD226 (Jeong and Park, 2020). Recent work suggests PD-1 signaling may predominantly inhibit CD28 over TCR (Hui et al., 2017), sparking new interest in combination/fusion anti-PD-1 checkpoint therapies (Waite et al., 2020; Liu et al., 2021). Ongoing work in our group suggests PD-1 attenuates both TCR and CD28 signaling to protect early expanding T cells from RICD.

### Metabolic programming

Metabolic plasticity shapes all phases of the adaptive immune response, which likely influences temporal changes in RICD sensitivity that impact effector T cell dynamics in different environments. Many aspects of T cell metabolism offer enticing avenues for further investigation, including potential relationships to co-signaling receptors. PD-1 is able to upregulate fatty acid oxidation from a glycolytic state in activated T cells through the PI-3K and MEK/Erk pathways, possibly accounting for the longevity of PD1+ T cells (Patsoukis et al., 2015; Kalia et al., 2021). More work is needed to understand how co-signaling molecules influence signaling networks (e.g., PI-3K, mTOR) at the nexus of cellular metabolism and apoptosis regulation, especially during clonal expansion (Patsoukis et al., 2017; Saravia et al., 2020). Aberrent RICD may also be a feature of specific immune disorders involving genetic dysregulation of these pathways, such as activated phosphatidylinositol 3-kinase delta (PI3Kδ) syndrome (APDS). Accelerated glycolysis and enhanced Fas-mediated apoptosis compromises CD8^+^ CM T cell formation in a mouse model of APDS, which could be interpreted as a state of RICD hypersensitivity (Cannons et al., 2021). Direct effects of various metabolites on apoptosis sensitivity also demands further study. For example, memory and effector T cells differ in their assimilation of acetate, with acetate dampening TCR signaling in memory T cells during restimulation, pointing to a metabolic shift that may influence RICD survival (Balmer et al., 2020). Nutrient deprivation, hypoxia and high lactate levels within the tumor microenvironment are known to debilitate T cell function (Sugiura and Rathmell, 2018), but ramifications for T cell survival in an antigen-dense environment remain unclear.

### T cell subsets

Human T cell heterogeneity poses challenges in trying to understand cell survival dynamics, considering that naïve, effector, and memory cell lineages possess distinct transcriptomic and metabolic profiles that affect their response to antigen (Gerriets et al., 2015; Golubovskaya and Wu, 2016). Therefore, RICD sensitivity will likely vary in cells originating from different populations—as an example, EM cells are uniquely susceptible to Fas and TCR-induced apoptosis *ex vivo* relative to other subsets (Ramaswamy et al., 2011). Work from our lab suggests human CD8^+^ effectors derived from EM and CM compartments differ in RICD sensitivity, mirroring differences in CWID (Larsen et al., 2017b). We have also studied heightened resistance of Treg cells to RICD compared to conventional CD4^+^ T cells (Katz et al., 2018), wherein differential sensitivity of Th subsets is already well-established (see above). These disparities in RICD susceptibility among subsets undoubtedly have clinical significance in understanding immunodeficiencies and autoimmune conditions, and optimizing adoptive T cell therapies for cancer treatment.

### Unbiased “omics” screens

Recent advancements in bioinformatics and large-scale genomic/proteomic screens provide exciting opportunities to harness unbiased approaches for investigating regulators of programmed cell death in T cells. As an example, a recent whole genome CRISPR screen identified early B cell factor 4 (EBF4) as a mediator of FAS/CD95-induced apoptosis resistance in activated T cells (Kubo et al., 2022). Similar screens should be employed to discover hitherto unknown molecular determinants of TCR-induced apoptosis. Next-generation single cell sequencing technologies also offer a valuable tool for interrogating heterogeneous T cell pools and identifying distinct effector cell states associated with RICD susceptibility or resistance (Bradley et al., 2018; Szabo et al., 2019).

## Conclusion

Successful adaptive immune responses follow an elegant cycle of activation, expansion and contraction to control or eliminate foreign pathogens. Self-regulatory programs that govern T cell dynamics are vital to ensuring a sufficient but controlled response, preventing the body from enduring T cell hyperproliferation and dangerous immunopathology. RICD is one key “propriocidal” mechanism for controlling T cell survival capacity when antigen and IL-2 are present. While terminally differentiated T cells readily succumb to RICD, recently activated T cells remain relatively resistant to RICD to facilitate clonal expansion. A greater molecular understanding of temporal changes to T cell apoptosis sensitivity, including RICD, will hopefully illuminate ways to correcting dysregulated immune homeostasis in human diseases including autoimmune disease, immunodeficiencies, and cancer.

## Figures and Tables

**TABLE 1:** Human immune disorders associated with defective T cell apoptosis.

Disease	Inheritance	Genetic defect(s)	Apoptosis defect(s)	Pathology
X-linked lymphoproliferative disease (XLP-1)	XL	*SH2D1A* (SAP)	RICD	EBV-induced FIM, HLH, hypogammaglobulinemia, lymphomas
Autoimmune lymphoproliferative syndrome (ALPS)	AD (incomplete)	*FAS/CD95* (germline/somatic), *FASL*, *CASP10*	FAS-included apotosis, RICD (partial)	Splenomegaly, lymphadenopathy, ↑ DNTs, autoimmune cytopenias, lymphomas
ALPS-like	AD	*STAT5B*	RICD (TEM cells)	Splenomegaly, lymphadenopathy, ITP, hypogammaglobulinemia, elevated TEM cells
RAS-associated leukoproliferative disease (RALD)	AD	*NRAS*, *KRAS* (somatic)	CWID, RICD	Splenomegaly, lymphadenopathy, monocytosis, IDNTs, hypergammaglobulinemia, progression to JMML

AD, autosomal dominant; DNTs, double negative T cells; EBV, Epstein-Barr virus; FIM, fulminant infectious mononucleosis; HLH, hemophagocytic lymphohistiocytosis; ITP, immune thrombocytopenic purpura; JMML, juvenile myelomonocytic leukemia.
